# Rapamycin Protects Against Peritendinous Fibrosis Through Activation of Autophagy

**DOI:** 10.3389/fphar.2018.00402

**Published:** 2018-04-20

**Authors:** Wei Zheng, Yun Qian, Shuai Chen, Hongjiang Ruan, Cunyi Fan

**Affiliations:** Department of Orthopaedics, Shanghai Jiao Tong University Affiliated Sixth People’s Hospital, Shanghai, China

**Keywords:** rapamycin, peritendinous fibrosis, autophagy, transforming growth factor beta, cell cycle, extracellular matrix

## Abstract

Dysregulation of autophagy plays a pivotal role in fibrosis in multiple organs. However, the role of autophagy in peritendinous fibrosis is not well understood. Here, we hypothesize that autophagy plays a protective role in preventing adhesion formation. In a rat model of tendon injury, we observed dysregulated autophagy during excessive extracellular matrix deposition. Pharmacological induction of autophagy by rapamycin markedly alleviated the severity of peritendinous fibrosis *in vivo*. In NIH/3T3 fibroblasts and tenocytes, transforming growth factor β1 (TGF-β1) markedly activated myofibroblasts and increased collagen synthesis. Addition of rapamycin activated autophagy, reduced collagen synthesis, and suppressed myofibroblast activation. *In vitro* experiments also showed that rapamycin decreased cell proliferation and increased the number of cells arrested in G_0_/G_1_ phase. However, following pretreatment with the autophagy inhibitor 3-methyladenine (3-MA), rapamycin was unable to repress the fibrotic changes induced by TGF-β1. Autophagy related protein 5 (*Atg5*) RNA interference in fibroblasts also abolished the protective effects of rapamycin *in vitro*. In conclusion, our results point to rapamycin as a potential treatment strategy in the prevention of peritendinous fibrosis after tendon injury.

## Introduction

Peritendinous fibrosis is a critical issue in the field of orthopedics, and results in tendon gliding dysfunction and limitations in daily life (Manske, 1988). Studies suggest that peritendinous adhesion formation is an abnormal healing process, characterized by activation of cell proliferation and excessive extracellular matrix (ECM) deposition (Katzel et al., 2011; Juneja et al., 2012). Myofibroblasts, key effectors in fibrotic disorders, contribute to ECM deposition, tissue contracture, and functional impairment (Darby et al., 2016). Although many studies have focused on the molecular mechanism of peritendinous fibrosis, the initiation of this process remains unclear. Previous studies have demonstrated abnormal cytokine release in adhesive tendon tissues, including transforming growth factor (TGF)-β, fibroblast growth factor-2, connective tissue growth factor, and vascular endothelial growth factor; among these, TGF-β is the most extensively studied (Juneja et al., 2012; Wynn and Ramalingam, 2012; Meng et al., 2015). TGF-β expression is increased in fibrotic diseases, such as pulmonary, kidney, and liver fibrosis, and systemic sclerosis (Ding et al., 2014; Sosulski et al., 2015; Musso et al., 2016; Manetti et al., 2017). Several studies have demonstrated that TGF-β is the most powerful regulator of myofibroblast phenotype, stimulating the conversion of quiescent fibroblasts to α-smooth muscle actin (α-SMA)-positive (differentiated) myofibroblasts (Desmoulière et al., 1993; Rønnov-Jessen and Petersen, 1993). It has been shown that TGF-β induces the development of fibrosis through the canonical Smad2/3 signaling pathway and non-canonical signaling pathways, such as the extracellular signal–regulated kinases 1/2 (ERK1/2) and mammalian target of rapamycin (mTOR) pathways (Hayashida et al., 2003; Li et al., 2009). In addition, TGF-β stimulation breaks the balance of the cell cycle, which is important for cell homeostasis, and promotes cell proliferation (Pattarayan et al., 2018).

Autophagy is a highly conserved biological process that provides an adaptive response under various stimuli, including starvation, endoplasmic reticulum stress, hypoxia, and oxidative stress (Lee et al., 2012; Hale et al., 2013). The process of autophagy includes engulfing unnecessary elements (including misfolded proteins and non-functional organelles) in double-membrane autophagosomes, which then fuse with lysosomes to form autolysosomes and ultimately degrade. This process can occur in bulk or can be highly selective and is controlled by the coordinated action of various autophagy-related genes (*Atg*), including *Atg6, Atg5, Atg13, Atg16, Atg7*, and *Atg8*; notably, dysregulation of one gene can markedly impair autophagy. mTOR serves as a core component of two protein complexes, mTOR complex 1 (mTORC1) and mTOR complex 2 (mTORC2) (Lipton and Sahin, 2014). Upon activation, mTORC1 phosphorylates Atg 13, prevented the formation of ULK1 kinase complex, and inhibited autophagy activation. Rapamycin, discovered more than 30 years ago, has been shown to inhibit the activation of mTORC1, and induce autophagy (Hartford and Ratain, 2007).

Previous studies have demonstrated dysregulated autophagy activity in various physiological and pathophysiological states, such as cancer, aging, and inflammatory diseases. More recently, researchers have also highlighted the protective role of autophagy in the fibrosis of various tissues, such as kidney, lung, and heart (Luciani et al., 2010; Kim et al., 2012; Li et al., 2016; Liu et al., 2016). Blockage of autophagy results in increased TGF-β levels, fibroblast to myofibroblast differentiation (FMD), and collagen synthesis (Ding et al., 2014). Recently, researchers have reported the correlation between autophagy and cell cycle. Double immunofluorescence staining for microtubule-associated proteins 1A/1B light chain 3B (LC3B) and cyclins shows that autophagy is preferentially induced in the G1 and S phases of the cell cycle, and is inhibited in the G2 and M phases (Tasdemir et al., 2007). During starvation, autophagy is required for mitotic exit and arrest in the G1/G0 quiescent state (An et al., 2014). Similarly, Atg5 overexpression is reported to attenuate collagen synthesis by inhibiting the G2/M cell cycle arrest (Li et al., 2016).

Although autophagy has been associated with fibrosis of different tissues, the effect of autophagy on peritendinous fibrosis is still unknown. In the present study, for the first time, we explored the relationship between autophagy and progression of peritendinous fibrosis. Furthermore, we evaluated whether rapamycin could prevent peritendinous fibrosis through activation of autophagy. Our results show that collagen deposition significantly increases when autophagy decreases. Importantly, induction of autophagy by rapamycin effectively suppresses cell proliferation, inhibits myofibroblast activation, decreases ECM production, and ultimately alleviates peritendinous fibrosis.

## Materials and Methods

### Reagents and Antibodies

Dulbecco’s modified Eagle medium (DMEM), fetal bovine serum (FBS), penicillin-streptomycin (PS), and trypsin were purchased from Gibco (Grand Island, NY, United States). TGF-β1 was purchased from R&D systems (Minneapolis, MN, United States). Rapamycin was from Selleck (Houston, TX, United States). 3-MA was from Sigma-Aldrich (St Louis, MO, United States). Antibodies against Col1, Col3, α-SMA, and TGF-β1 were from Abcam (Cambridge, United Kingdom). Antibodies against phosphorylated p-mTOR, LC3A/B, p62, Atg5, and glyceraldehyde 3-phosphate dehydrogenase (GAPDH) were purchased from Cell Signaling Technology (Danvers, MA, United States).

### Cell Culture and Treatments

Mouse NIH/3T3 fibroblasts were purchased from the Cell Bank of Type Culture Collection, Chinese Academy of Sciences (Shanghai, China). For primary tenocyte isolation, tendons were firstly cut into 1 mm^3^ pieces, digested with 0.15% collagenase NB4 (SERVA, Germany) for 2 h at 37°C, filtered through cell meshes (Corning, NY, United States), and centrifugated at 1,000 rpm for 5min. The cell pellets were then resuspended in the culture medium. Fibroblasts and primary tenocytes were cultured in DMEM supplemented with 10% FBS and 1% PS, in an atmosphere of 5% CO_2_ at 37°C. The medium was replaced every 2–3 days, and fibroblasts and tenocytes were passaged when they reached 80% confluence. Cells were treated with TGF-β1 (2 ng/mL) and/or rapamycin (500 nM). To inhibit autophagy, cells were pretreated with 3-MA (2 mM) for 2 h. siRNA targeting *Atg5* or scramble siRNA (**Table 1**) were purchased from GenePharma (Shanghai, China). Fibroblasts were transfected using Lipofectamine 2000 (Invitrogen, Carlsbad, CA, United States). In brief, fibroblasts were incubated overnight, at 37°C, with a transfection solution containing opti-MEM (Invitrogen), the siRNAs and Lipofectamine 2000.

**Table 1:** The Atg5 siRNA sequences for mouse.

Gene	Sequences (5′ → 3′)	
mus atg5 siRNA-1	Sense	GCUUCGAGAUGUGUGGUUUTT
	Antisense	AAACCACACAUCUCGAAGCTT
mus atg5 siRNA-2	Sense	CCAUCAACCGGAAACUCAUTT
	Antisense	AUGAGUUUCCGGUUGAUGGTT

### Animal Experiments

Sprague-Dawley rats were purchased from the Shanghai Laboratory Animal Company (Shanghai, China). All animal studies were approved by the Animal Care and Use Committee of the Shanghai Jiao Tong University Affiliated Sixth People’s Hospital. Eight-week-old male rats were used in the experiments. For the TI model, rats underwent resection of the superficial flexor tendon of the plantar. We exposed and transected the deep flexor tendon, and repaired the deep flexor tendon with 6-0 sutures. For SO rats, an incision was made medially over the skin of the plantar and subsequently sutured. To activate autophagy, rats were injected with rapamycin (2 mg/kg/d) intraperitoneally. Control rats were injected with the same volume of dimethyl sulfoxide. Rats were sacrificed 3 weeks after operation. Tendon tissues were harvested and stored at -80°C or fixed in 4% paraformaldehyde (PFA) or 2.5% glutaraldehyde.

### Hydroxyproline (Hyp) Content

Hydroxyproline content in tendon tissues was quantified as previously described (Li et al., 2015). In brief, tendon tissues were homogenized and hydrolyzed in concentrated HCl for 2 h at 110°C. Then, 0.5 mL chloramine-T reagent was added before incubation for 20 min at room temperature (RT), followed by incubation with 0.5 mL Ehrlich’s Reagent at 65°C for 15 min. Absorbance was measured at 550 nm, and the Hyp content was shown as mg of Hyp per mL.

### Histological Staining, Immunohistochemistry, and Immunofluorescence

Tendon tissues sections embedded in paraffin were stained with H&E or Masson staining solutions. The histological severity of tendon adhesion was assessed as follows: (1) no adhesion formation; (2) less than 33% of tendon surface; 3, 33–66% of tendon surface; and 4, more than 66% of the tendon surface (Jiang et al., 2014; Chen et al., 2017).

Immunohistochemical or immunofluorescence staining of tendon tissues or cells was performed according to standard procedures. In brief, tendon tissue sections (5 μm thickness) or cells were fixed in 4% PFA, washed with phosphate-buffered saline (PBS), incubated with primary antibodies overnight at 4°C and then, with horseradish peroxidase (HRP)- or fluorophore-conjugated secondary antibodies for 60 min at RT. Cell proliferation was analyzed using a Ki67 labeling and detection kit (Sigma). Images were viewed using a microscope and analyzed using the ImageJ software (National Institute of Health, Bethesda, MD, United States).

### Confocal Microscopy and TEM

GFP-RFP-LC3-transfected NIH/3T3 fibroblasts were exposed to different treatments for 24 h. After treatment, cells were washed with PBS and examined under a confocal microscope (Leica SP8, Germany). For TEM examination, cells were fixed in 2.5% glutaraldehyde overnight at 4°C, post-fixed with 1% osmium tetroxide for 1 h at RT, dehydrated, and embedded. The number of AVs per cell was examined under a transmission electron microscope (Hitachi, Japan).

### Quantitative PCR (qPCR) and Western Blotting

Tendon tissues or cells were homogenized in TRIzol reagent (Invitrogen). Total RNA were extracted and 1 μg of total RNA was reverse-transcribed into cDNA in a reaction primed with oligo-dT primers (Takara, Kusatsu, Japan) according to the manufacturer’s instructions. qPCR was performed using SYBR Green Premix Ex Taq polymerase (Takara). GAPDH was used as an internal reference for normalization. Primer sequences are shown in **Table 2**.

**Table 2:** The RT-PCR primer sequences for mouse.

Gene	Primer sequences (5′ → 3′)	
mus Col1	RealF	GCTCCTCTTAGGGGCCACT
	RealR	CCACGTCTCACCATTGGGG
mus Col3	RealF	CTGTAACATGGAAACTGGGGAAA
	RealR	CCATAGCTGAACTGAAAACCACC
mus α-SMA	RealF	GTCCCAGACATCAGGGAGTAA
	RealR	TCGGATACTTCAGCGTCAGGA
mus Atg5	RealF	TGTGCTTCGAGATGTGTGGTT
	RealR	GTCAAATAGCTGACTCTTGGCAA
mus GAPDH	RealF	AGGTCGGTGTGAACGGATTTG
	RealR	TGTAGACCATGTAGTTGAGGTCA

Tendon tissues or cells were homogenized and lysed in radioimmunoprecipitation buffer containing protease and phosphatase inhibitors. The protein concentration was determined with a bicinchoninic acid assay (Thermo). Twenty micrograms of protein were loaded onto 10 to 15% gels for sodium dodecyl sulfate polyacrylamide gel electrophoresis, and electrotransferred onto polyvinylidene difluoride membranes after separation. Membranes were incubated with primary and secondary antibodies, and visualized using the enhanced chemiluminescent solution according to the standard protocol. GAPDH was used for normalization. Density analysis was performed using the ImageJ software.

### Cell Proliferation Analysis

Cell viability was assessed using the CCK8 kit (Dojindo, Kumamoto, Japan). Cells were cultured for 24 h at a density of 3 × 10^3^ per well in a 96-well plate and then treated with TGF-β1 and/or rapamycin. After 48 h, cell viability was assessed by incubating each well with 100 μl of a 10% CCK8 solution in medium for 2 h at 37°C and measuring the absorbance at 450 nm in a microplate reader.

EdU staining (Ribobio, Guangzhou, China) was used to assess proliferative cells. Cells were treated for 48 h and incubated with 50 μM EdU reagent overnight at 37°C. Then, cells were fixed in 100 μl of a 4% PFA solution for 30 min and treated with 0.5% Triton X-100 for 10 min at RT. After washing with PBS, cells were incubated with 100 μl of 1× Apollo reaction mixture for 30 min and stained with 100 μl of 1× Hoechst 33342 for 30 min. Cells were visualized using a fluorescence microscope.

### Flow Cytometry Analysis

After the indicated treatment for 48 h, cells were harvested with trypsin and washed with PBS. For apoptosis analysis, Annexin V-fluorescein isothiocyanate (FITC) and propidium iodide (PI) were incubated with the cell suspension for 30 min at RT. For cell cycle distribution analysis, cells were harvested, washed with ice-cold PBS, and fixed with 70% ethanol overnight at -20°C. Cells were resuspended in PBS, treated with RNase A, and stained with PI for 30 min. Apoptosis and cell cycle distribution were analyzed using a flow cytometer (Beckman Coulter, Brea, CA, United States).

### Statistical Analysis

Quantitative data are shown as the mean ± the standard deviation (SD), whereas qualitative data are shown as proportions. All experiments were performed at least three independent times. *P*-values < 0.05 were considered statistically significant. Statistical analyses were performed using the SPSS 21.0 software (Chicago, IL, United States).

## Results

### Autophagy Inversely Correlates With Fibrotic Changes After Injury-Induced Peritendinous Fibrosis

**FIGURE 1:**
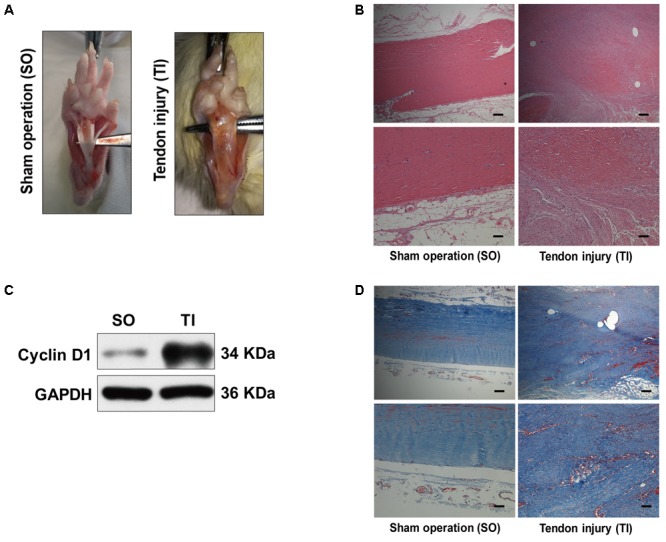
Peritendinous adhesion formation after tendon injury. **(A)** Representative images of macroscopic observation of deep flexor tendon of the plantar at 21 days after sham operation (SO) or tendon injury (TI). **(B)** Representative images of H&E staining of peritendinous tissues. Scale bar (upper): 250 μm. Scale bar (lower): 100 μm. **(C)** Representative images of western blot analysis of Cyclin D1 expression in SO and TI rats. **(D)** Representative images of masson staining of peritendinous tissues at 21 days after SO or TI rats. Scale bar (upper): 250 μm. Scale bar (lower): 100 μm. *n* = 5 in each group.

To examine autophagy in the development of peritendinous fibrosis, we collected tendon tissues from rats after tendon injury (TI). Tendon tissues from rats receiving a sham operation (SO) were used as control. Twenty-one days after SO, macroscopic observation showed that the tendon was smooth (**Figure 1A**). However, 21 days after TI, the tendon was wrapped in hyperplastic soft tissue and sharp dissection was required to separate the two. Hematoxylin and eosin (H&E) staining showed, in the SO group, that the cells were sparse and aligned. In contrast, we observed excessive proliferative and irregular cells in the TI group (**Figure 1B**). Cyclin D1 is a regulatory subunit: it binds to and activates cyclin-dependent kinase 4 (CDK4), therefore regulating the G_1_ to S phase transition and promoting cell proliferation. Western blot showed that the expression of Cyclin D1 was relatively low in tendon tissues from SO rats, and strongly increased in peritendinous adhesive tissues from TI rats (**Figure 1C**). Additionally, Masson staining revealed significantly increased peritendinous ECM deposition after TI (**Figure 1D**).

**FIGURE 2:**
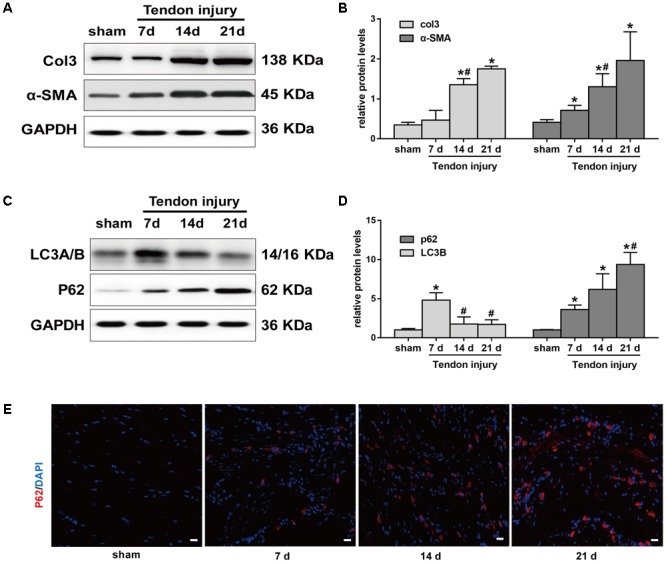
Autophagy and fibrotic changes after tendon injury. **(A)** Representative images of western blot analysis of Col3 and α-SMA in sham operation (SO) and tendon injury (TI) rats. **(B)** Densitometry of Col1 and α-SMA expression. **(C)** Representative images of western blot analysis of LC3A/B and p62 in different groups. **(D)** Densitometry of Col1 and α-SMA expression. **(E)** Representative images of immunofluorescent staining for p62 in different groups. Scale bar: 20 μm. *n* = 3 in each group. The data are shown as the mean ± SD. ^∗^*P* < 0.05 compared with the SO group. #*P* < 0.05 compared with 7 days after TI.

Meanwhile, we evaluated Col3 and α-SMA levels at 7, 14, and 21 days, two crucial fibrotic markers, in the two groups. Compared with the SO rats, the expression of Col3 significantly increased in TI rats at 14 days, and peaked at 21 days (**Figures 2A,B**). Similarly, the expression of α-SMA gradually increased after TI and peaked at 21 days. Western blotting analyses showed that LC3B levels were relatively low in tendon tissue from SO rats (**Figures 2C,D**). In contrast, in TI rats, we observed that the expression of LC3B peaked at 7 days, and markedly decreased at 14 and 21 days. p62 is another marker of autophagy involved in the recognition of aggregated proteins. Western blotting showed low expression of p62 in SO rats (**Figures 2C,D**). In TI rats, protein levels of p62 increased after surgery and peaked at 21 days, indicating decreased degradation of p62. Similarly, immunofluorescence staining for p62 showed a significant increase in the number of p62-positive cells in tissues from TI rats at 21 days compared with SO rats (**Figure 2E**). These results show that the development of peritendinous fibrosis accelerates when autophagy diminishes.

### Rapamycin Treatment Alleviates the Severity of Peritendinous Fibrosis

**FIGURE 3:**
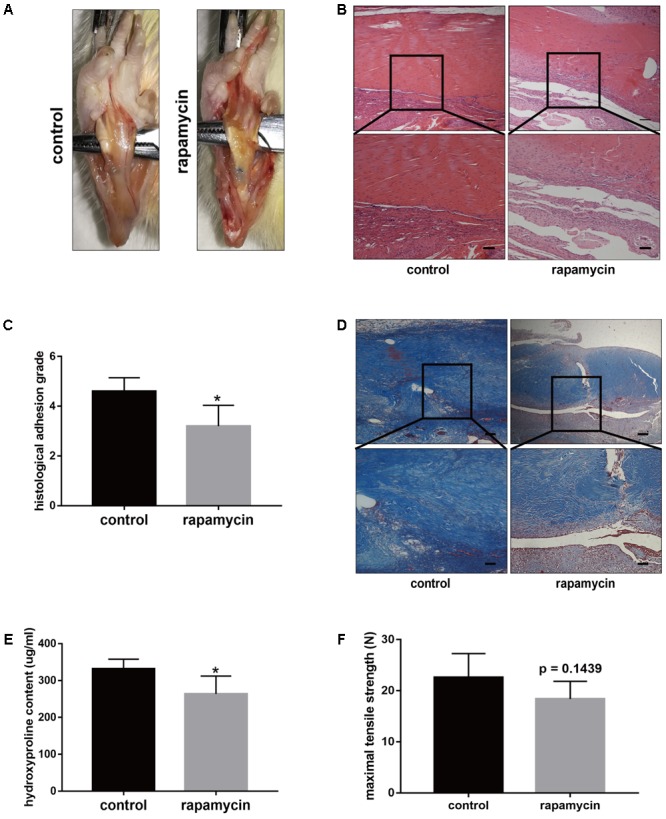
Rapamycin administration prevents peritendinous fibrosis after tendon injury. **(A)** Representative images of macroscopic observation of deep flexor tendon of plantar in control and rapamycin-treated group. **(B)** Representative images of H&E staining of peritendinous tissues. Scale bar (upper): 250 μm. Scale bar (lower): 100 μm. **(C)** Histological evaluation of adhesion grade in different groups. **(D)** Representative images of masson staining for collagen in different groups. Scale bar (upper): 250 μm. Scale bar (lower): 100 μm. **(E)** Hydroxyproline (Hyp) content in different groups. **(F)** Maximal tensile strength (N) of deep flexor tendon in different groups. *n* = 5 in each group. The data are shown as the means ± SD. ^∗^*P* < 0.05 compared with the control group.

The above data indicated excessive cell proliferation and ECM deposition after TI operation. To demonstrate whether activation of autophagy prevents the progression of peritendinous fibrosis, rats were treated with rapamycin (2 mg/kg/d) by intraperitoneal injection. Macroscopic observation showed that the amount of adhesive tissue was reduced upon rapamycin treatment (**Figure 3A**). Additionally, H&E staining revealed that excessive cell proliferation around the tendon was alleviated in rapamycin-treated rats (**Figure 3B**). Compared with DMSO-treated rats, the histological grade of the adhesion was lower in the rapamycin-treated rats (**Figure 3C**). Additionally, Masson staining showed decreased collagen deposition after rapamycin treatment (**Figure 3D**). Similarly, hydroxyproline (hyp) content was decreased by rapamycin treatment (**Figure 3E**). However, when we measured the maximal tensile strength, we found that rats treated with rapamycin showed a slightly reduction in strength, but the difference with the control rats was not significant (18.4 ± 3.1 N vs. 22.6 ± 4.2 N, *p* = 0.1439) (**Figure 3F**).

**FIGURE 4:**
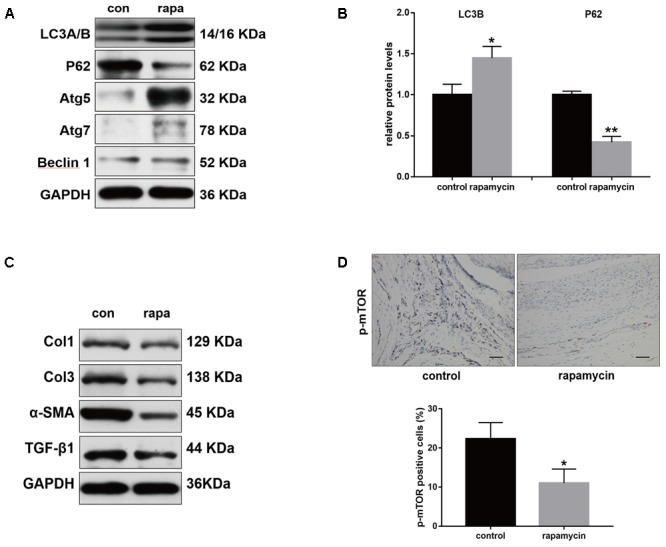
Rapamycin regulates expression of fibrotic genes after tendon injury. **(A)** Representative images of western blot analysis of LC3A/B, p62, Atg5, Atg7, and Beclin 1 after different treatments. **(B)** Densitometry of LC3A/B and p62 expression. **(C)** Representative images of western blot analysis of fibrotic gene expressions after different treatments. **(D)** Representative images of immunohistochemical staining and quantitative analysis for p-mTOR after different treatments. Scale bar: 50 μm. *n* = 5 in each group. The data are shown as the means ± SD. ^∗^*P* < 0.05 and ^∗∗^*P* < 0.01 compared with the control group.

Furthermore, we examined the autophagy activity after different treatment. Western blot showed that rapamycin did not induced autophagy in SO rats (**Supplementary Figure S1**). In TI rats, rapamycin treatment significantly increased expression of LC3B and degradation of p62 in peritendinous tissues (**Figures 4A,B**). Rapamycin administration markedly decreased the expression of Col1, Col3, and α-SMA (**Figure 4C**). The level of TGF-β1, a strong profibrotic factor, was also suppressed by rapamycin treatment. Additionally, immunohistochemical staining for phosphorylated (p)-mTOR showed that rapamycin markedly reduced the activation of the mTOR signaling pathway (**Figure 4D**).

**FIGURE 5:**
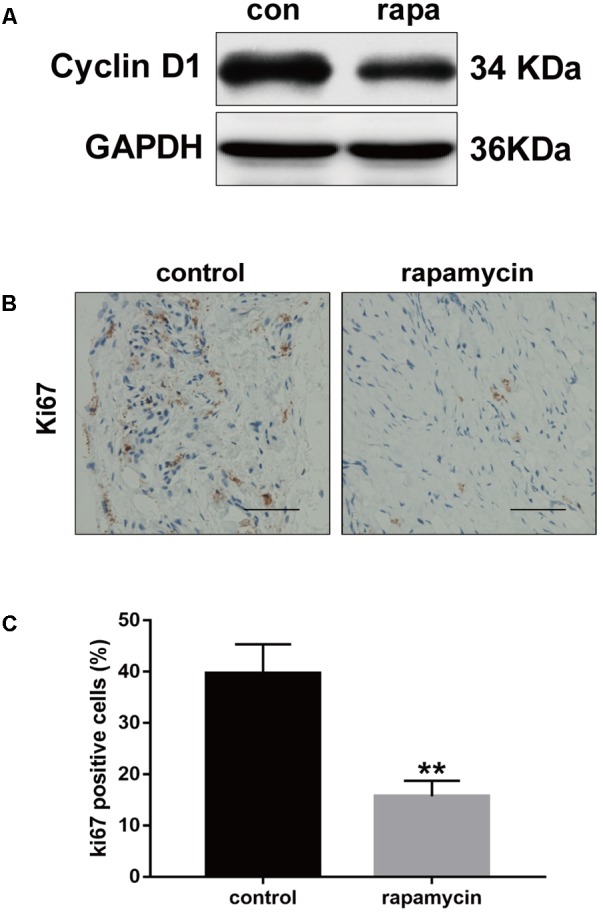
Rapamycin inhibits excessive proliferation after tendon injury. **(A)** Representative images of western blot analysis of Cyclin D1 after DMSO or rapamycin treatment. **(B)** Representative images of Ki67 staining and **(C)** quantitative analysis after different treatments. Scale bar: 50 μm. *n* = 5 in each group. The data are shown as the means ± SD. ^∗∗^*P* < 0.01 compared with the control group.

Excessive cell proliferation is suggested to play an important role in fibrotic diseases. Therefore, we investigated the effect of autophagy activation on cell proliferation. Cyclin D1 protein levels were markedly reduced following rapamycin treatment (**Figure 5A**). Ki67 is a cellular marker of cell proliferation (Scholzen and Gerdes, 2000). We observed a high number of proliferative cells in adhesive peritendinous tissues (**Figures 5B,C**). Contrarily, rapamycin treatment markedly decreased the number of proliferative cells. Taken together, these results show that rapamycin significantly induces autophagy, prevents excessive cell proliferation, and reduced ECM synthesis, indicating that autophagy plays a protective role against fibrosis after tendon injury.

### Induction of Autophagy Decreases TGF-β1-Induced Fibrogenesis *in Vitro*

**FIGURE 6:**
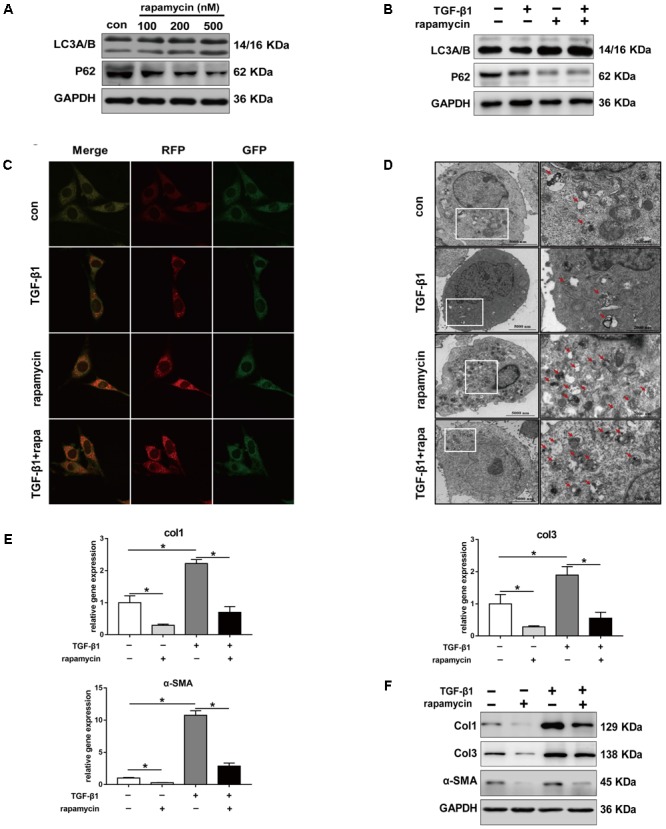
Rapamycin regulates fibrotic gene expression in NIH/3T3 fibroblasts. **(A)** Representative images of western blot analysis of LC3A/B and p62 protein levels after different concentration of rapamycin treatment for 24 h. **(B)** Representative images of western blot of LC3A/B and p62 levels after TGF-β1 (2 ng/ml) and/or rapamycin (500 nM) treatment for 24 h. **(C)** Representative images of fibroblasts stably transfected with GFP-RFP-LC3 were treated with TGF-β1 and/or rapamycin. Images were obtained by confocal microscope. Green and red-positive puncta indicate autophagosomes; green-negative, red positive puncta indicate autophagolysosomes. **(D)** Representative images of fibroblasts were treated with TGF-β1 and/or rapamycin, then autophagic vesicles (AVs) were analyzed by transmission electron microscope. **(E)** mRNA and **(F)** protein levels of Col1, Col3, and α-SMA after treated with TGF-β1 and/or rapamycin. Data are shown as the mean ± SD of three independent experiments. ^∗^*P* < 0.05.

Given the role of TGF-β1 in fibrotic changes, we evaluated the effect of TGF-β1 on autophagy by treating NIH/3T3 fibroblasts with TGF-β1 and/or rapamycin for 24 h. Fibroblasts treated with increasing concentration of rapamycin showed gradually enhanced autophagy (**Figure 6A**). Western blot analysis showed that, in fibroblasts treated with TGF-β1 (2 ng/mL), addition of rapamycin (500 nM) also significantly induced autophagy (**Figure 6B**). Next, we transfected NIH/3T3 cells with lentiviral particles encoding the green fluorescent protein (GFP)-red fluorescent protein (RFP)-LC3B fusion protein and monitored autophagy under different treatments using confocal microscopy. Early autophagosomes appear yellow because they display both green and red fluorescence, whereas autophagolysosomes display only red fluorescence. After treatment with rapamycin for 24 h, we observed that the number of both the yellow and red puncta increased obviously (**Figure 6C**). Transmission electron microscopy (TEM) also revealed more autophagic vesicles (AVs) containing engulfed organelles in rapamycin-treated fibroblasts (**Figure 6D**). Further we assessed the effect of rapamycin on fibrotic changes. Fibroblasts treated with TGF-β1 showed significantly increased mRNA levels of *Col1* and *Col3*, whereas addition of rapamycin inhibited this effect (**Figure 6E**). Western blot assays confirmed the inhibitory effect of rapamycin on collagen synthesis (**Figure 6F**). TGF-β1 has been proven to strongly induce FMD *in vitro*. Consistent with this, we observed a marked increase in *α-SMA* expression following TGF-β1 treatment, whereas rapamycin abolished the effect of TGF-β1 (**Figures 6E,F**).

**FIGURE 7:**
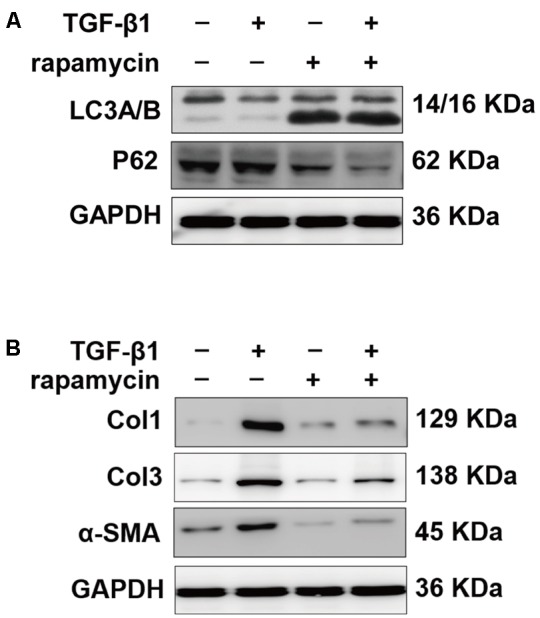
Rapamycin regulates fibrotic gene expression in primary tenocytes. **(A)** Representative images of western blot of LC3A/B and p62 levels after TGF-β1 (2 ng/ml) and/or rapamycin (500 nM) treatment for 24 h. **(B)** Representative images of western blot analysis of Col1, Col3, and α-SMA after different treatments. All experiments were performed three independent times.

We also treated primary tenocytes with TGF-β1 and/or rapamycin. Western blot analysis showed that rapamycin significantly induced upregulation of LC3B and degradation of p62, indicating autophagy activation (**Figure 7A**). TGF-β1 treatment upregulated protein levels of Col1, Col3, *and* α-SMA in tenocytes, whereas addition of rapamycin markedly attenuates the effect of TGF-β1 (**Figure 7B**). Collectively, these findings suggest that rapamycin induces autophagy and prevents fibrogenesis by inhibiting collagen synthesis and myofibroblast activation.

### Induction of Autophagy Regulates the Proliferation and Cell Phase Arrest Induced by TGF-β1 *in Vitro*

**FIGURE 8:**
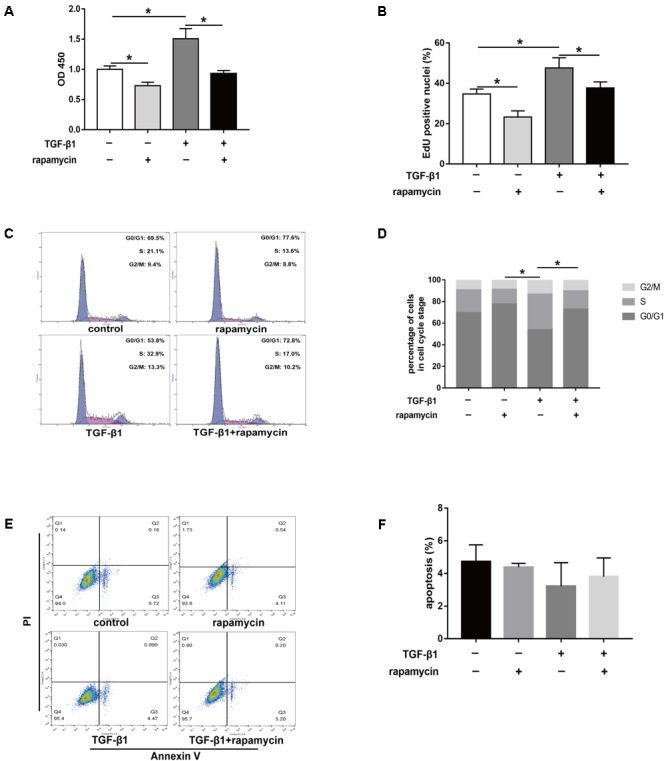
Rapamycin treatment suppresses fibroblast proliferation. **(A)** CCK8 analysis of cell viability after treated with TGF-β1 and/or rapamycin for 48 h. **(B)** Quantitative analysis of proliferative fibroblasts by EdU staining after different treatments for 48 h. **(C)** Representative images of flow cytometry and **(D)** quantitative analysis of cell cycle distribution after different treatments for 48 h. **(E)** Representative images of flow cytometry and **(F)** quantitative analysis of apoptotic fibroblasts after different treatments for 48 h. Data are shown as the mean ± SD of three independent experiments. ^∗^*P* < 0.05.

Excessive cell proliferation has been suggested to be an important pathological feature during fibrogenesis. First, we used the cell counting kit 8 (CCK8) to assess fibroblast viability under different treatment conditions for 48 h. We observed that fibroblast viability significantly increased after TGF-β1 treatment (**Figure 8A**). However, addition of rapamycin attenuated the effects of TGF-β1. Similarly, 5-ethynyl-2′-deoxyuridine (EdU) assay also revealed an increased percentage of proliferative fibroblasts after TGF-β1 treatment, whereas the percentage of proliferative cells decreased following rapamycin treatment (**Figure 8B**). Next, we assessed the cell cycle distribution using flow cytometry. TGF-β1 treatment for 48 h induced a marked increase in the percentage of fibroblasts arrested in S and G_2_/M phase compared with control fibroblasts (**Figures 8C,D**). Addition of rapamycin significantly increased the proportion of cells arrested in G_0_/G_1_ phase. Additionally, we performed flow cytometry, to detect apoptotic cells following the different treatments. We found few apoptosis in control or TGF-β1-treated fibroblasts and treatment with rapamycin did not alter the percentage of apoptotic fibroblasts (**Figures 8E,F**).

**FIGURE 9:**
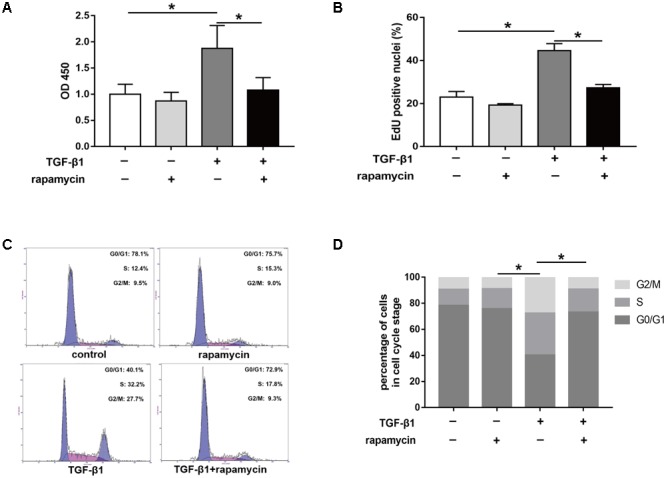
Rapamycin treatment suppresses tenocyte proliferation. **(A)** CCK8 analysis of cell viability after treated with TGF-β1 and/or rapamycin for 48 h. **(B)** Quantitative analysis of proliferative tenocytes by EdU staining after different treatments for 48 h. **(C)** Representative images of flow cytometry and **(D)** quantitative analysis of cell cycle distribution after different treatments for 48 h. Data are shown as the mean ± SD of three independent experiments. ^∗^*P* < 0.05.

Furthermore, we assessed the effect of rapamycin on tenocyte proliferation. We obtained similar results to those in fibroblasts. CCK8 showed that rapamycin significantly suppressed the TGF-β1-induced increase in cell viability (**Figure 9A**). EdU staining revealed that rapamycin reduced the number of proliferative tenocytes induced by TGF-β1 (**Figure 9B**). However, rapamycin treatment alone did not inhibit tenocyte proliferation compared with the control group. In cell cycle analyses, we found that addition of rapamycin to TGF-β1-treated tenocytes significantly induced G_0_/G_1_ phase arrest compared with TGF-β1 alone (**Figure 9C,D**).

Collectively, these findings suggest that rapamycin inhibits cell proliferation by inducing a G_0_/G_1_ cell phase arrest.

### Inhibition of Autophagy Abolishes the Protective Role of Rapamycin Against Peritendinous Fibrogenesis *in Vitro*

**FIGURE 10:**
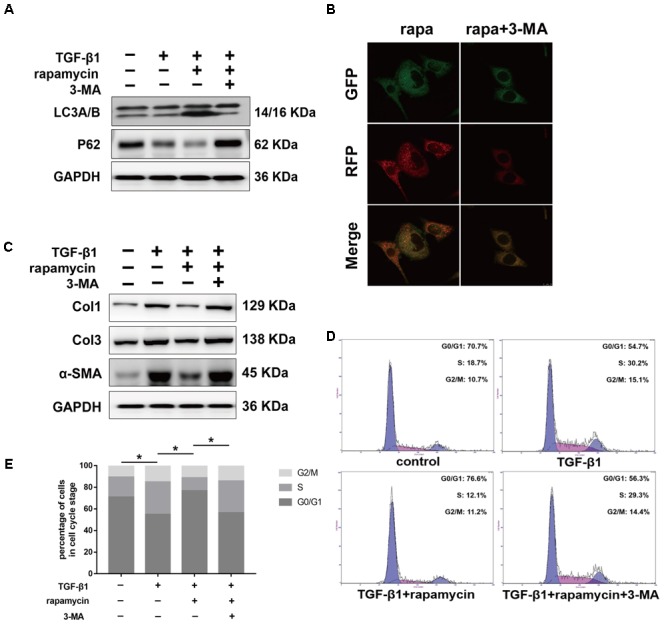
Autophagy inhibitor 3-MA abolishes the protective effects of rapamycin in fibroblasts. Fibroblasts were pretreated with 3-MA (2 mM) for 2 h before further treatments. **(A)** Representative images of western blot analysis of LC3A/B and p62 after different treatments. **(B)** Representative images of fibroblasts stably transfected with GFP-RFP-LC3 were treated with 3-MA and rapamycin. **(C)** Representative images of western blot analysis of Col1, Col3, and α-SMA after different treatments. **(D)** Representative images of flow cytometry and **(E)** quantitative analysis of cell cycle distribution after different treatments. The data are shown as the means ± SD of three independent experiments. ^∗^*P* < 0.05.

Next, we examined whether the protective effects of rapamycin against fibrogenesis were autophagy-dependent. Specifically, we inhibited autophagy in fibroblasts and tenocytes using a pharmacological inhibitor, 3-MA. Western blotting confirmed that 3-MA inhibited LC3B accumulation and p62 degradation (**Figure 10A**). Additionally, in fibroblasts transfected with GFP-RFP-LC3B and pretreated with 3-MA, we observed that rapamycin was unable to induce the formation of yellow or red puncta, confirming inhibition of autophagy (**Figure 10B**). Western blot showed that pretreatment with 3-MA abolished the effect of rapamycin on TGF-β1-induced collagen synthesis and α-SMA expression (**Figure 10C**). Next, we investigated cell cycle progression after inhibition of autophagy. Rapamycin significantly suppressed the effect of TGF-β1 on cell cycle by inducing G_0/_G_1_ arrest. However, in fibroblasts pretreated with 3-MA, the effect of rapamycin on cell cycle was abolished and the proportion of cells arrested in S and G_2_/M was similar to that observed upon treatment with TGF-β1 alone (**Figures 10D,E**).

**FIGURE 11:**
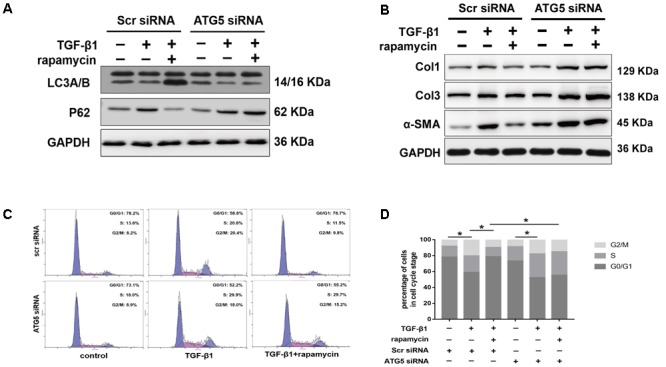
Autophagy inhibition by *Atg5* siRNA abolishes the protective effects of rapamycin in fibroblasts. Fibroblasts were transfected with Scramble or *Atg5* siRNA before further treatments. **(A)** Representative images of western blot analysis of LC3A/B and p62 after treated with TGF-β1 and/or rapamycin for 24 h. **(B)** Representative images of western blot analysis of Col1, Col3, and α-SMA after different treatments. **(C)** Representative images of flow cytometry and **(D)** quantitative analysis of cell cycle distribution after different treatments. The data are shown as the means ± SD of three independent experiments. ^∗^*P* < 0.05.

We interfered with *Atg5* expression in NIH/3T3 fibroblasts by RNA interference. Upon *Atg5* knockdown, addition of rapamycin failed to induce the expression of LC3B or the degradation of p62 (**Figure 11A**). Additionally, cells transfected with *Atg5* siRNA or scramble siRNA showed no significant difference in the levels of fibrotic genes (**Figure 11B**). In similar conditions, western blot assays showed that rapamycin failed to attenuate TGF-β1-induced Col1, Col3, and α-SMA expression. In fibroblasts transfected with scramble siRNA, rapamycin prevented the increase of cells arrested in S and G_2_/M phase (**Figures 11C,D**). However, after *Atg5* knockdown, rapamycin did not exert this effect.

**FIGURE 12:**
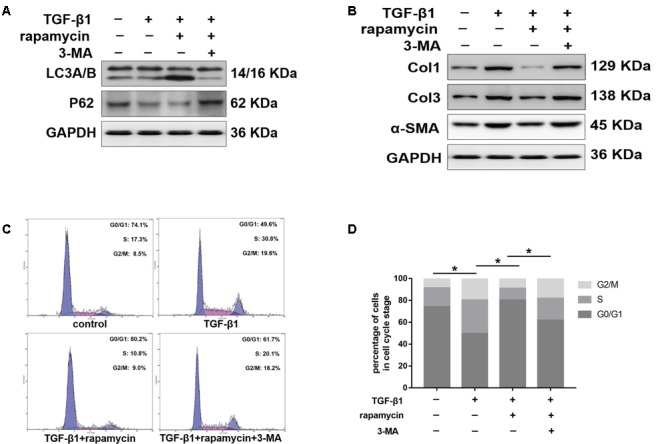
3-MA abolishes the protective effects of rapamycin in tenocytes. Tenocytes were pretreated with 3-MA (2 mM) for 2 h before treated with TGF-β1 and/or rapamycin. **(A)** Representative images of western blot analysis of LC3A/B and p62 after different treatments. **(B)** Representative images of western blot analysis of fibrotic genes after different treatments. **(C)** Representative images of flow cytometry and **(D)** quantitative analysis of cell cycle distribution after different treatments. The data are shown as the means ± SD of three independent experiments. ^∗^*P* < 0.05.

We further treated primary tenocytes with 3-MA. Rapamycin was unable to induce autophagy in tenocytes pretreated with 3-MA (**Figure 12A**). Meanwhile, rapamycin did not prevent the upregulation of fibrotic genes induced by TGF-β1 (**Figure 12B**). In addition, although rapamycin promoted G0/G1 phase arrest in tenocytes, pretreatment with 3-MA significantly suppressed this effect (**Figures 12C,D**).

In summary, these data suggest that rapamycin attenuates collagen synthesis and induces G_0_/G_1_ arrest through an autophagy-dependent pathway. All the original images of western blot are shown in **Supplementary Figure S2**.

## Discussion

Tissue fibrosis is considered an abnormal wound healing process that occurs after stimulus, during which the balance between collagen synthesis and degradation is broken. Fibrogenesis in different tissues has similar pathological features, including the inflammatory response, myofibroblast activation, and subsequent excessive ECM deposition, and functional tissue degeneration (Wynn and Ramalingam, 2012). Peritendinous fibrosis, a unique pathological tissue fibrosis, mostly occurs after tendon injury, tendon surgery, and joint immobilization. Several mechanisms have been implicated in the modulation of adhesion formation, including the regulation of the inflammatory response (Chen et al., 2017), and the TGF signaling pathway (Jiang et al., 2014; Zheng et al., 2017). The present study, for the first time, to our knowledge, examines the relationship between autophagy and peritendinous fibrosis, and demonstrates protective roles of autophagy in fibrogenesis. Reduced autophagy occurred in parallel with enhanced fibrotic changes after tendon injury. Pharmacological activation of autophagy by rapamycin alleviated severity of peritendinous fibrosis. In fibroblasts and tenocytes, rapamycin markedly reduced ECM synthesis and induced G_0_/G_1_ arrest, however, inhibition of autophagy by 3-MA or Atg5 siRNA abolished the protective effects of rapamycin.

Existing evidences have demonstrated that autophagy dysfunction correlates with pathological fibrosis. In a renal fibrosis model, researchers showed that Col1 accumulation was accompanied by the gradual decrease of autophagy after UUO (Li et al., 2016). Interference of atg5 aggravated Ang II induced accumulation of collagen in rat cardiac fibroblasts (Liu et al., 2016). Furthermore, in mice with genetic ablation of autophagy-related genes, including *atg5, atg4b, LC3*, and *beclin 1*, inflammatory responses and pathological fibrotic changes were exacerbated (Ding et al., 2014; Cabrera et al., 2015; Lodder et al., 2015; Li et al., 2016). In the present study, by constructing a deep flexor tendon injury model, we found significant peritendinous fibrosis formation at 21 days. Western blot analysis showed that both the protein level of LC3B and p62 were relatively low in the SO group, indicating low activity of autophagy in normal tendon tissues. After TI surgery, LC3B expression temporarily increased during the initial stages and rapidly reduced, whereas the protein level of p62 gradually increased and peaked at 21 days. Meanwhile, collagen synthesis markedly increased since the autophagy began to decline. These findings highlighted the potential negative correlation between autophagy activity and fibrogenesis after tendon injury. Hence, we propose autophagy as a protective mechanism against peritendinous fibrosis. In previous studies, pharmacological activation of autophagy showed protective effects against fibrogenesis. By co-treatment with a known activator of autophagy, trifluoperazine, the col1 expression induced by TGF-β1 was significantly reduced in mouse mesangial cells (Kim et al., 2012). In mice with unilateral ureteral obstruction (UUO), rapamycin treatment attenuated the tubulointerstitial fibrosis, however, proximal tubular epithelial cell-specific deletion of *Atg5* abolished the protective effects of rapamycin and exacerbated renal fibrosis (Li et al., 2016). Overexpression of MiR-449a significantly activated autophagy and reduced the extent and severity of lung fibrosis induced by silica through targeting Bcl2 (Han et al., 2016). In a kidney fibrosis model, induction of autophagy was shown to significantly inhibit TGF-β expression (Ding et al., 2014). In our study, TI rats treated with rapamycin showed increased activity of autophagy. Through macroscopic and histological evaluation, we found that the severity of fibrosis was significantly alleviated by rapamycin. TGF-β1 is an important profibrotic factor, which strongly promotes the synthesis of ECM. Our present study demonstrated that rapamycin significantly suppressed the production of TGF-β1 after TI, thereby decreasing the collagen synthesis. *In vitro* studies also showed that rapamycin inhibited TGF-β1 induced collagen synthesis in fibroblasts and tenocytes, in accordance with the *in vivo* experiments. By confocal analysis, we also noticed that addition of TGF-β1 induced slightly increase in positive puncta. We think this may be because addition of TGF-β1 was a stimulus and it temporarily induced mild autophagy. It is worth noting that ECM is the main component of tendon, and sometimes prevention of peritendinous fibrosis adversely affects the tendon healing (Jiang et al., 2014). In our study, we found that the maximal tensile strength was slightly lower in rapamycin-treated rats, but the difference was not statistically significant.

Excessive cell proliferation is a distinctive biological process in fibrotic diseases and the interconnection between autophagy-related gene expression and cell proliferation and apoptosis has been shown. Romero found that idiopathic pulmonary fibrosis fibroblasts showed resistance to apoptosis, likely mediated by persistent mTOR activation (Romero et al., 2016). Li revealed that *Atg5* interference aggravated G_2_/M arrest in proximal tubular epithelial cells (Li et al., 2016). In *beclin 1* heterozygous-deficient mice, decreased autophagy resulted in increased cellular proliferation (Qu et al., 2003). Existing evidences have shown that rapamycin inhibited tumor cell proliferation by promoting G_1_ phase arrest (Decker et al., 2003; Mateo-Lozano et al., 2003) and promoted apoptosis (Hosoi et al., 1999; Romero et al., 2016). During peritendinous fibrosis, there is a significant pathological accumulation of active cells. In the present study, we found no significant difference in apoptosis after rapamycin treatment. In our animal experiments, ki67 staining showed that excessive cell proliferation was attenuated by rapamycin after TI. In addition, rapamycin also reduced the expression level of Cyclin D1. *In vitro* experiments demonstrated that rapamycin significantly inhibited fibroblast and tenocyte proliferation. By further analysis of the cell cycle distribution, addition of rapamycin induced G_0_/G_1_ phase arrest. These findings suggested that rapamycin inhibited the G_1_ to S transition.

Furthermore, we performed *in vitro* experiments to demonstrate whether rapamycin inhibited peritendinous fibrosis through an autophagy-dependent pathway. First, we treated fibroblasts or tenocytes with 3-MA, which blocks autophagosome formation via the inhibition of class III PI3K, and found that, in these conditions, rapamycin was not able to prevent the collagen synthesis and myofibroblast activation induced by TGF-β1. The regulatory effect of rapamycin on cell cycle was also abolished by 3-MA. Atg5 is an E3 ubiquitin ligase which is necessary for autophagy owing to its role in autophagosome elongation. When we interfered with *Atg5* expression in fibroblasts, similarly, the protective effects of rapamycin were also abolished after *Atg5* interference. These findings suggest that the protective role of rapamycin on peritendinous fibrosis depends on autophagy activation.

In summary, our study indicates that autophagy is a determining factor in the progression of peritendinous fibrosis. Our data show that autophagy dysregulation is involved in adhesion formation. Rapamycin markedly activates autophagy and alleviates fibrosis by decreasing collagen synthesis, suppressing FMD, and promoting the G_0_/G_1_ cell phase arrest. Our findings indicate that rapamycin is a potential protective mechanism for preventing the progression of peritendinous fibrosis.

## Author Contributions

WZ: designed the study, performed the experiments, and drafted the manuscript. YQ: performed the experiments and analyzed the data. SC: collected and analyzed the data. HR: interpreted the data and drafted the manuscript. CF: designed the research, interpreted data, and approved the final version of manuscript.

## Conflict of Interest Statement

The authors declare that the research was conducted in the absence of any commercial or financial relationships that could be construed as a potential conflict of interest.

## Supplementary Material

FIGURE S1 Autophagy activity after shame operation. Representative images of western blot analysis of LC3A/B and p62 expression levels after DMSO/rapamycin treatment in shame operation rats. *n* = 3 in each group.

FIGURE S2 Original images of western blot.
