# Supplementary figures and images for: Rapamycin Protects Against Peritendinous Fibrosis Through Activation of Autophagy
Source: Front Pharmacol. 2018 Apr 20;9:402. doi: 10.3389/fphar.2018.00402 (PMC5921906; doi:10.3389/fphar.2018.00402)

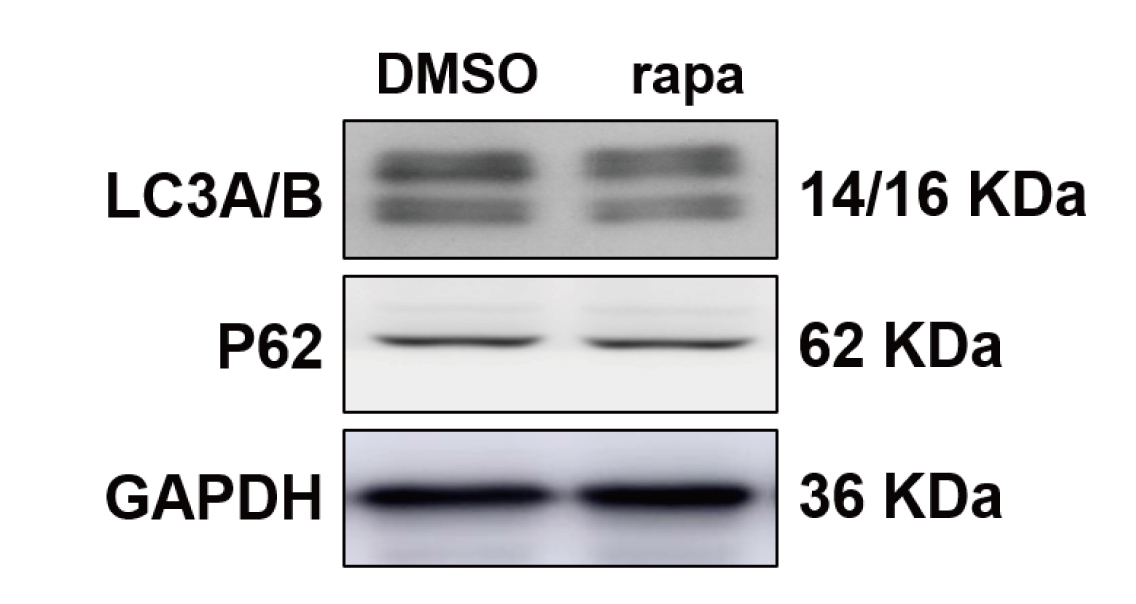

Supplement: FIGURE S1 — Autophagy activity after shame operation. Representative images of western blot analysis of LC3A/B and p62 expression levels after DMSO/rapamycin treatment in shame operation rats. n = 3 in each group. [file Image_1.jpg]

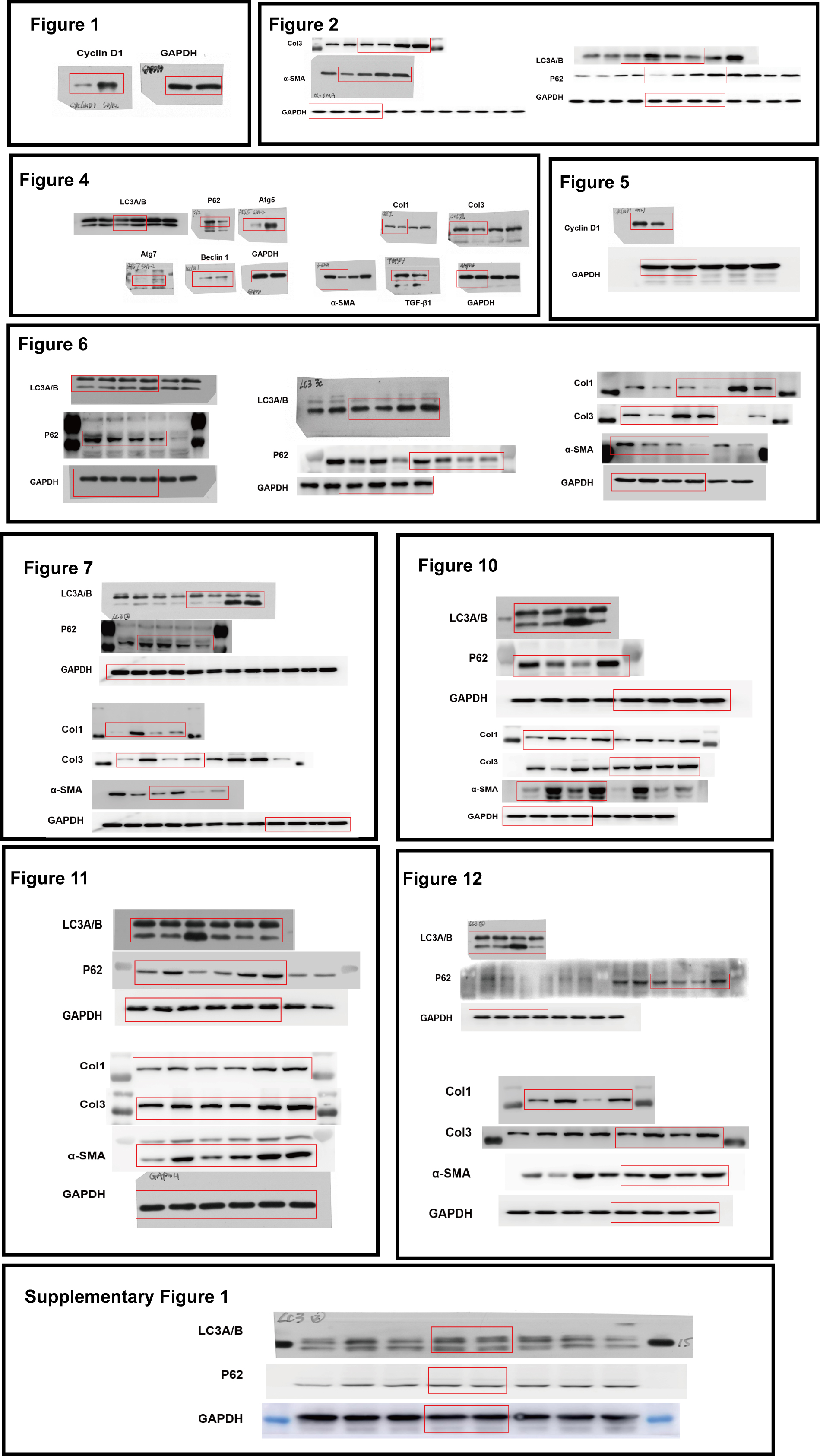

Supplement: FIGURE S2 — Original images of western blot. [file Image_2.jpg]
